# Moderate binding of villin headpiece protein to C_3_N_3_ nanosheet reveals the suitable biocompatibility of this nanomaterial

**DOI:** 10.1038/s41598-023-41125-1

**Published:** 2023-08-23

**Authors:** Yuqi Luo, Zonglin Gu, Jose Manuel Perez-Aguilar, Weihua Liao, Yiwen Huang, Yanbo Luo

**Affiliations:** 1 Department of Gastrointestinal and Hepatobiliary Surgery, Shenzhen Longhua District Central Hospital, No. 187, Guanlan Road, Longhua District, Shenzhen, 518110 Guangdong China; 2 College of Physical Science and Technology, Yangzhou University, Jiangsu, 225009 China; 3 School of Chemical Sciences, Meritorious Autonomous University of Puebla (BUAP), 72570 University City, Puebla Mexico; 4 Department of Radiology, Guangzhou Nansha District Maternal and Child Health Hospital, No. 103, Haibang Road, Nansha District, Guangzhou, 511457 Guangdong China; 5 Department of Emergency, Nansha Hospital, Guangzhou First People’s Hospital, Guangzhou, Guangdong China

**Keywords:** Computational biophysics, Nanoscale biophysics

## Abstract

Since its recent successful synthesis and due to its promising physical and chemical properties, the carbon nitrite nanomaterial, C_3_N_3_, has attracted considerable attention in various scientific areas. However, thus far, little effort has been devoted to investigating the structural influence of the direct interaction of this 2D nanomaterial and biomolecules, including proteins and biomembranes so as to understand the physical origin of its bio-effect, particularly from the molecular landscape. Such information is fundamental to correlate to the potential nanotoxicology of the C_3_N_3_ nanomaterial. In this work, we explored the potential structural influence of a C_3_N_3_ nanosheet on the prototypical globular protein, villin headpiece (HP35) using all-atom molecular dynamics (MD) simulations. We found that HP35 could maintain its native conformations upon adsorption onto the C_3_N_3_ nanosheet regardless of the diversity in the binding sites, implying the potential advantage of C_3_N_3_ in protecting the biomolecular structure. The adsorption was mediated primarily by vdW interactions. Moreover, once adsorbed on the C_3_N_3_ surface, HP35 remains relatively fixed on the nanostructure without a distinct lateral translation, which may aid in keeping the structural integrity of the protein. In addition, the porous topological structure of C_3_N_3_ and the special water layer present on the C_3_N_3_ holes conjointly contributed to the restricted motion of HP35 via the formation of a high free energy barrier and a steric hindrance to prevent the surface displacement. This work revealed for the first time the potential influence of the 2D C_3_N_3_ nanomaterial in the protein structure and provided the corresponding in-depth molecular-level mechanism, which is valuable for future applications of C_3_N_3_ in bionanomedicine.

## Introduction

Since their initial discovery, carbon-based nanomaterials (CBNs), including C_60_, carbon nanotube, and graphene, have been attracting notable attention from different scientific fields, due to their prominent physical and chemical properties^1–7^. Driven by their excellent mechanical, optical, and electrical properties, the applications of CBN in biomedicine has considerably increase, including those related to gene delivery, optical imaging, and nanotherapeutics^8–15^. Nevertheless, from the multiple biomedical utilization, the nanotoxicology and biocompatibility of these materials remains a central concern and was established as an important element in the development of nanoscience and nanotechnology around the biomedical applications of CBN. The direct interaction of CBN materials and biomolecules might cause damage to the latter, resulting in the potential nanotoxicity of the former. For example, graphene nanosheet had displayed potential toxicity to the prototypical water-soluble protein villin headpiece (HP35), through disruption of its secondary and tertiary. The perturbation of the structural integrity of HP35 was mainly caused by the strong π–π stacking interaction between aromatic residues and the graphene sheet^16^. Along the same lines, single-walled carbon nanotubes could occupy the active site of the WW and SH3 protein domains hence competing with the ligand peptide and precluding the native receptor–ligand binding, which leads to the potential nanotoxicity. Considering the ubiquity and significance of such proteins in signaling and regulatory pathways, interactions between carbon nanotubes and the WW and SH3 domains arose concerns about the CBNs’ usage in biological systems^17–19^. Similarly to the behavior of carbon nanotubes, other nanomaterial including fullerenes and metallofullerenols, had also exhibited competitive properties with the native ligands for the ligand binding site of the WW and SH3 domains^16,20^. Furthermore, among the different CBNs, graphene was deemed to own the strongest observed capacity for disrupting protein structure in systems with various surface topologies^16^. Therefore, scientists usually utilized CBNs in vivo application via the surface passivation using different surface coatings and functionalizations^21–24^.

Beyond the aforementioned nanomaterials, carbon nitride, a family subtype of CBN consisting of a regular arrangement of carbon and nitrogen atoms, including C_3_N_4_^25^, C_2_N^26^, C_3_N^27,28^, and C_3_N_3_^29,30^, has recently become a research hotspot, due to their inherent electronic and optoelectronic properties. Based on their surprising electronic conductivity and optical property, scientists have successfully designed various devices using carbon nitrides, such as photoelectrical devices, sensors, field-effect transistor devices and so on^26,28,31^. Carbon nitrides were also verified holding catalytic activity for H_2_ evolution and oxygen reduction reaction^32–34^. Moreover, some carbon nitrides exhibited promising potential in biomedical applications. For instance, ultrathin graphitic-phase C_3_N_4_ (g-C_3_N_4_) nanosheets, prepared by a “green” liquid exfoliation pathway from bulk g-C_3_N_4_ in water, presented excellent properties such as (1) good stability in both acidic and alkaline solvent, (2) intensive photoabsorption and photoresponse, (3) excellent biocompatibility^35^. Meanwhile, the g-C_3_N_4_ single-layer quantum dot was exploited for fluorescence imaging of the cellular nucleus^36^. Through hybridization with gold nanoparticles, the g-C_3_N_4_ was produced as an electrochemiluminescence immunosensor^37^. The g-C_3_N_4_ was also used as a platform to achieve up-conversion nanoparticles for cancer photodynamic therapy^38^. Lastly, Zhou et al.^39^ used experimental and theoretical approaches to reveal that carbon nitride quantum dots can inhibit the aggregation of the microtubule-associated protein tau via a direct interaction, featuring a potential treatment for Alzheimer's disease.

The structure of the C_3_N_3_ material shares a similar porous nanostructure with C_3_N_4_ and thus might present comparable physical and chemical features that could be exploited in bionanomedicine. In this context, previous studies have reported the interaction between proteins and some carbon nitride nanomaterials, such as C_3_N_4_^40^, C_2_N^41^, and C_3_N^42^. However, there has been limited knowledge regarding the interaction of C_3_N_3_ with biomolecules, even though this type of information is pivotal to assessing the bio-effect of the C_3_N_3_ nanomaterial. In this regard, our investigations have previously shown that the HP35 protein can unfold upon adsorption to C_3_N nanosheet, wherein (1) the aromatic residues robustly adhere and rapidly move on the planar C_3_N sheet, (2) the fast movement of the aromatic residues subsequently open the hydrophobic core of HP35 exposing the aromatic residues forming the protein’s hydrophobic the core, and (3) the exposure of aromatic residues in the HP35 core further denature the entire structure. In stark contrast, the tertiary protein structure can stabilize on the C_2_N and C_3_N_4_ surfaces due to the preferential adsorption of positively charged residues nearby the pores of the C_2_N and C_3_N_4_ sheets and the aggregated water layer formed on the surface of the C_2_N and C_3_N_4_ systems. This type of interaction confines the lateral movement of the protein and finally aids to maintain the native protein structure. However, no information thus far discloses the potential influence of the C_3_N_3_ nanomaterial in the protein structure. Therefore, in this study, we probed the direct binding of a model water-soluble protein, HP35, to the C_3_N_3_ nanosheet using all-atom molecular dynamics (MD) simulations. We found that the HP35 protein maintained its tertiary structural integrity upon binding to C_3_N_3_, exhibiting no obvious changes in either its secondary or tertiary structures. Furthermore, we observed that the transverse migration of the HP35 protein on the C_3_N_3_ plane was almost restricted. A specific conformation of a positively charged residue on the N-rich hole was detected. Further analysis confirmed that the fixed binding pattern, attributed to the porous structure of C_3_N_3_ and the special water layer on C_3_N_3_, was pivotal in preventing the lateral migration of the protein. Our works revealed in detail the interaction basis between the C_3_N_3_ nanomaterial and a model protein, which might be correlated to the nanomaterial’s suitable biocompatibility at the molecular level.

## Result

**Figure 1:**
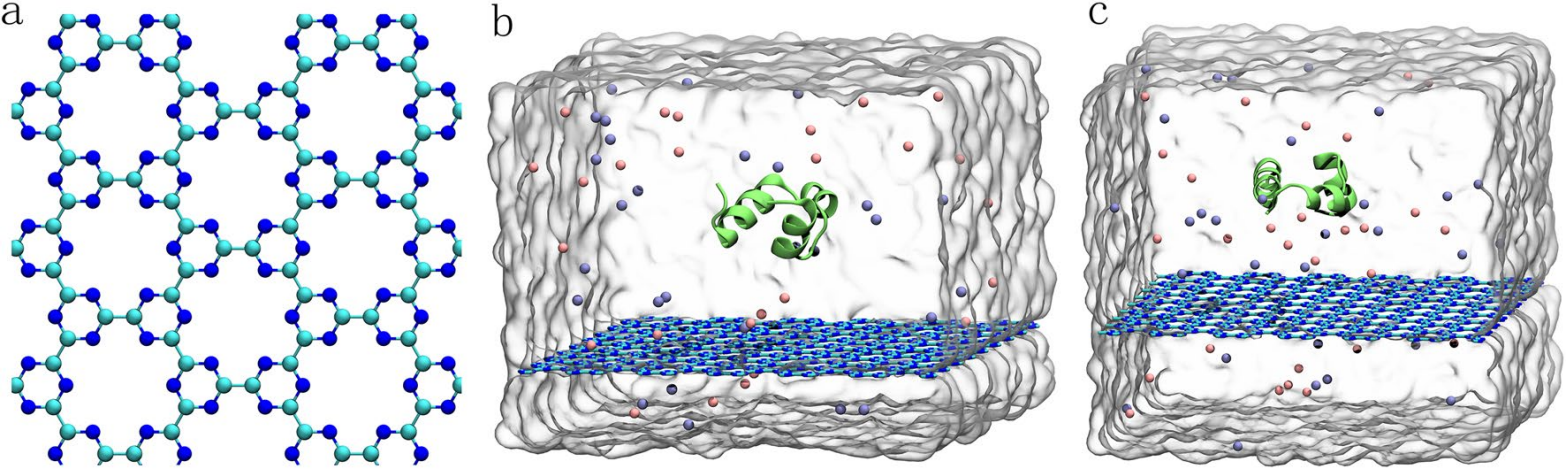
C_3_N_3_ structure and initial simulation setups. (**a**) In the C_3_N_3_ structure, the carbon and nitrogen atoms were shown with cyan and blue spheres, respectively. (**b**,**c**) The initial simulation setups. We rotated the HP35 protein to acquire two initial systems with the different sides facing the C_3_N_3_ (**b**, defined as sys1, and **c**, defined as sys2). The HP35 structure is displayed by lime ribbons. The C_3_N_3_ nanosheet is depicted by the stick representations. Na^+^ and Cl^-^ ions are exhibited with pink and iceblue spheres, respectively. The water box boundary of the setups was also shown by gray surfaces.

**Figure 2:**
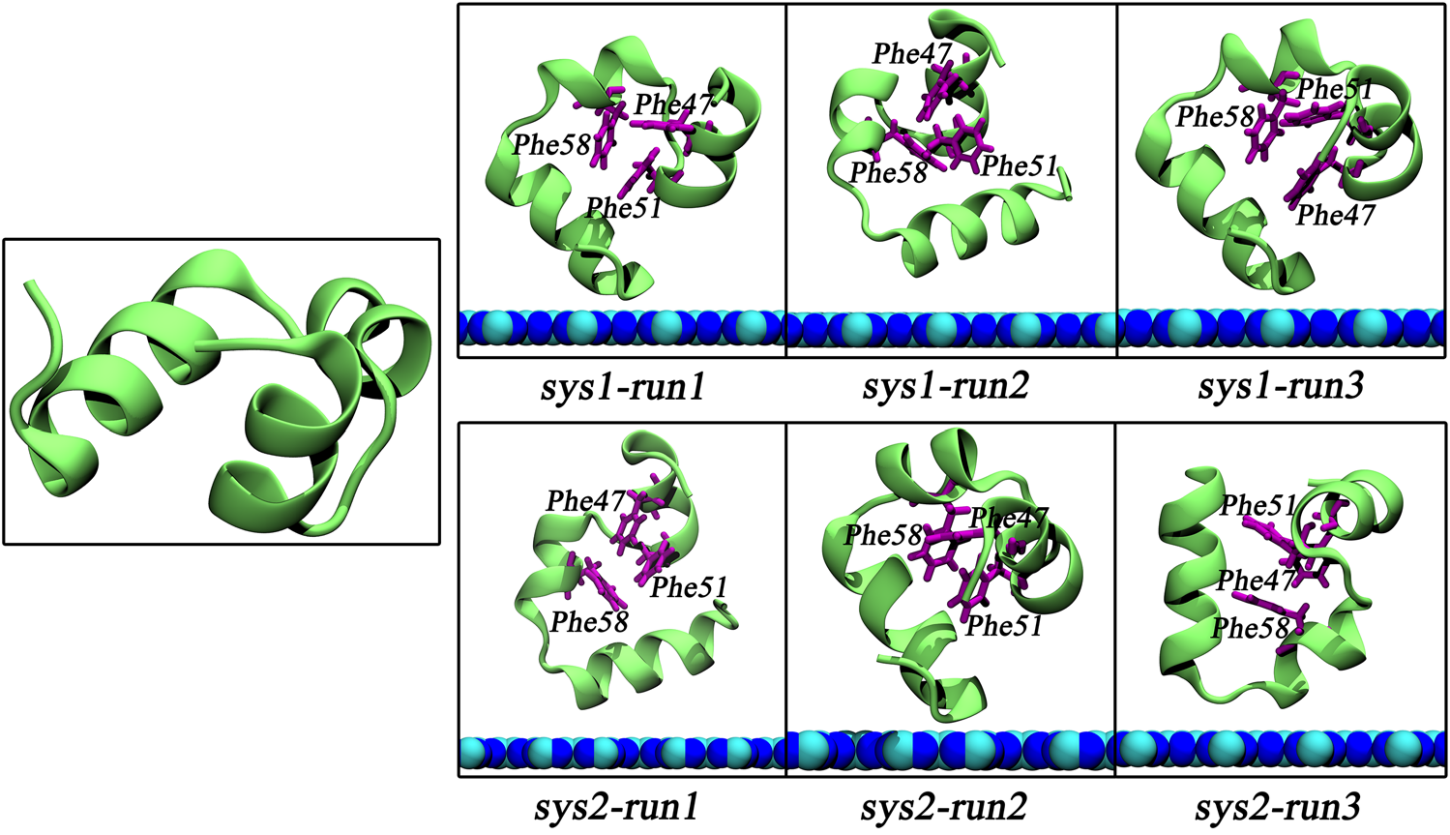
The HP35 structure before and after the binding event to the C_3_N_3_ surface. The left picture illustrated the initial structure of HP35. The six pictures in the right panels indicated the final conformations of HP35 binding to the C_3_N_3_ nanosheet. Three key aromatic residues (Phe47, Phe51, and Phe58), identified as the hydrophobic core of this protein, maintained the native conformation without significant changes.

The C_3_N_3_ nanosheet is a holey 2-dimensional nanomaterial comprised of ordered carbon and nitrogen atoms, where the holes are surrounded by six nitrogen atoms (Fig. 1a). This structure is similar to the structure of C_2_N and was successfully synthesized recently^29,30^. It has been demonstrated that C_2_N displayed a mild binding to biomolecules without perturbing the tertiary structure of the biomolecules^41,43^. However, to our knowledge, there was no information regarding the potential bio-effect of the C_3_N_3_ nanomaterial thus far. Hence, the present study, we investigated the interaction between a C_3_N_3_ nanosheet and a typical water-soluble protein to assess the potential effect of the nanomaterial on biomolecules and to underlie the molecular mechanism, prior to its exploitation in biomedical applications. We chose the prototypical HP35 protein as a model protein because its folding and unfolding dynamics have been extensively studied and featured general properties associated with common globular proteins despite its small size^44–47^. Two initial simulation systems were set up as shown in Fig. 1b,c, in which the HP35 protein was placed on the C_3_N_3_ nanosheet, yielding two different orientations (see more details in the Methods section). Each system was investigated by 200 ns-length MD simulations for three parallel trajectories. The final conformations were illustrated in Fig. 2. Clearly, the HP35 protein maintained its tertiary structure in the six systems: (1) the structure of the three α-helices in the six conformations remained the same as that in the initial conformation; (2) the interfacial binding protein positions also presented no obvious structural loss; (3) more importantly, the hydrophobic core formed by three aromatic residues, phenylalanine (Phe47, Phe51 and Phe58), maintained its native conformation and integrity in the protein core. Furthermore, structural alignments at the original and final frames in each trajectory, shown in Fig. S1, indicate that the HP35 retains adequate structural stability during the simulations, suggesting that the adsorption of HP35 on the C_3_N_3_ nanosheet does not alter its native tertiary structure. This result suggests an appropriate biocompatibility of the C_3_N_3_ nanomaterial. Therefore, the direct contact with the C_3_N_3_ nanosheet did not perturb the integrity of the HP35 3D-structure regardless of the location of the binding site.

**Figure 3:**
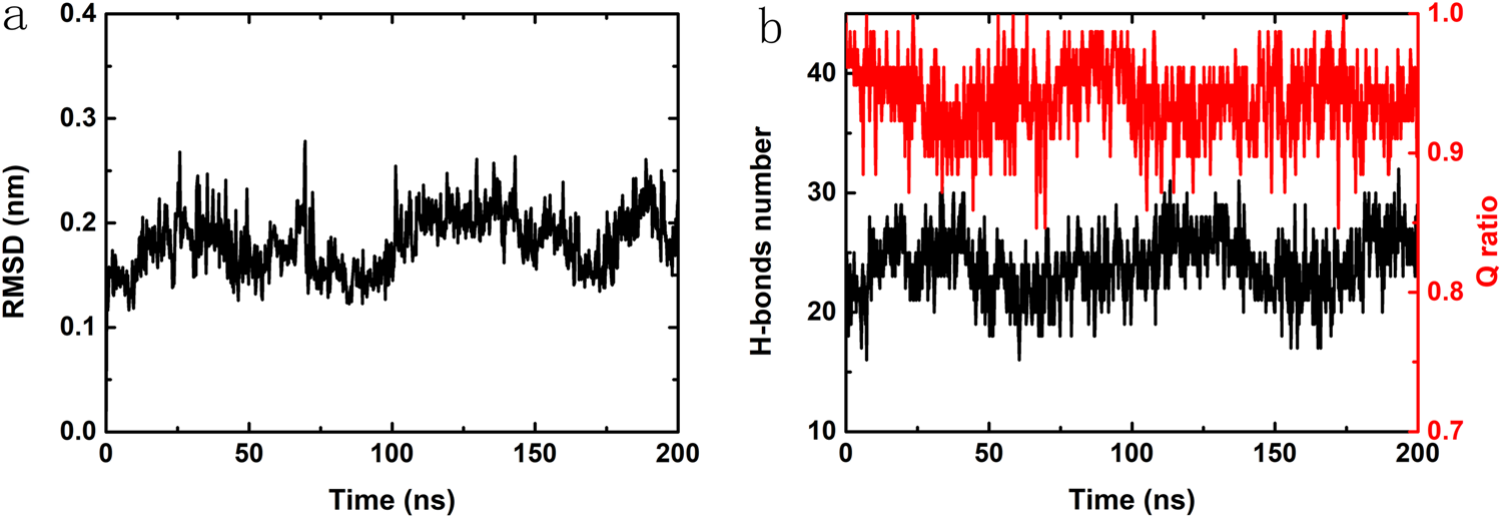
Structural analysis of the HP35 by choosing a representative trajectory. (**a**) The root-mean-square deviation (RMSD) of the heavy atoms of HP35 during the trajectory. (**b**) The hydrogen bond (H-bond) and Q ratio evolutions of HP35 during the trajectory are depicted in black and red, respectively.

To quantitatively evaluate the changes in the structure of HP35 upon binding to the C_3_N_3_ nanosheet, we calculated the root-mean-square deviation (RMSD) of the heavy atoms of HP35, the hydrogen bond number within HP35 as well as the Q value of the protein (as seen in Fig. 3). It was notable that the RMSD of heavy atoms exhibited a minor fluctuation around 0.2 nm, indicating that the HP35 just suffered a slight perturbation after interacting with the C_3_N_3_ nanosheet. Also, the hydrogen bond number within the HP35 also presented a similar tendency. Furthermore, a *Q* ratio, that is, the fraction of the remaining native contacts with respect to the simulation time, was quantified to estimate the changes in the tertiary structure of HP35. Here, *Q* was defined as the ratio of the total number of native contacts (using a distance cutoff of 6 Å) in the sampled structures to that identify the crystal structure (PDB accession code 1YRF), according to a previous protocol^48^. Similarly, the *Q* ratio was almost over 0.9 during the entire simulation, suggesting that HP35 maintained the contacts associated with the native tertiary structure without any detrimental alteration caused by the binding event. In addition, the calculations of other trajectories also verified the same conclusion (Figs. S2, S3). We also plotted the 2D-dimensional residue contact map of the HP35 at the initial and final frames of the simulation as shown in Fig. S4. Clearly, the contact map does not reflect a distinct difference within these two structures, which indicates that the HP35 keeps its entire 3D-structure during its adsorption onto the C_3_N_3_ surface. Our quantitative analyses further confirmed that the HP35 protein could be adsorbed onto the C_3_N_3_ nanosheet without exhibiting any significant structural alteration.

**Figure 4:**
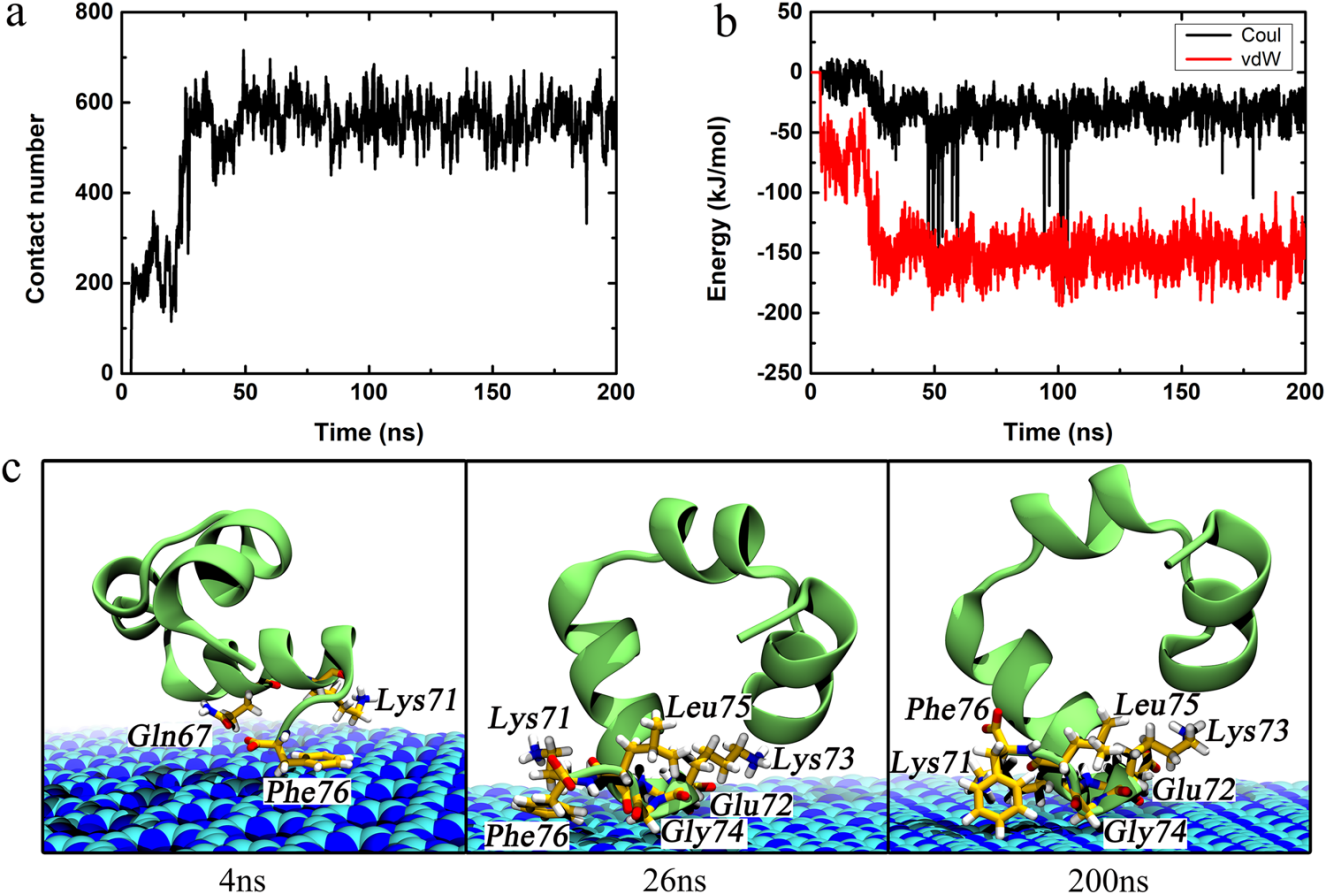
Interaction kinetics of the HP35 binding to the C_3_N_3_ nanosheet of sys1. (**a**) The contact number of the HP35 binding to the C_3_N_3_ nanosheet is depicted. (**b**) The interaction energies, the van der Waals (vdW) and the Coulombic (Coul) energies, between C_3_N_3_ and HP35 are shown. (**c**) The binding conformations in some key time points. The relevant amino acids were displayed by sticks and labeled with their residue names.

In order to dissect the underlying binding kinetics, we computed the atom contact number, interaction energies (including van der Waals (vdW) and Coulombic (Coul) energies) as well as depicted some snapshots by choosing a representative trajectory of the simulated system, sys1, as shown in Fig. 4. The contact number gradually increased with a concomitant decreased in the vdW energy term. Notably, the contact number curve was almost symmetrical to that profile of the vdW energy curve, implying that the vdW interaction dominated the binding of HP35 onto the C_3_N_3_ surface. Moreover, the vdW energy was much stronger than the Coulombic energy counterpart, further supporting the importance of the former interaction (although some sudden declines in the Coulombic energy were observed, see more discussion below). Based on these alterations in the contact number and interaction energies, we plotted three snapshots of the system in three key time points to describe the kinetic process more clearly. Early at t = 4 ns, the HP35 started its original contact from three residues on the same helix (one side of the protein chain), Gln67, Lys71, and Phe76. Meanwhile, the contact number and the vdW energy displayed sharp increments, with their values reaching 195.0 and − 61.1 kJ/mol. Following, the binding approached a transitory plateau/metastable state until 26 ns. At this point, the HP35 protein underwent a reorientation, resulting in the local loss of some of the initial contacting residues (Gln67) albeit with the formation of new interactions (Glu72, Lys73, and Gly74). This further contact is accompanied by an abrupt rise in the atom contact number (to 632) together with the strengthening of the vdW and Coulombic energies (vdW energy to − 156.1 kJ/mol; Coulombic energy to − 11.2 kJ/mol). From the 26 ns to the end of the simulation (200 ns), the adsorption reached an equilibrium state, holding the contact number and the vdW energy with limited fluctuations. The same analyses were also performed on a trajectory denominated sys2 (Fig. S5). The adsorption in this trajectory directly arrived at an equilibrium state once the HP35 protein contacted the C_3_N_3_ surface (at t = 19 ns). After 19 ns, the contact number and interaction energies exhibited small fluctuations due to the fine adjustment of the contact conformations of the interfacial residues (Lys71, Glu72, Lys73, Gly74, Leu75, and Phe76). Furthermore, to determine the contribution of each atom type on the C_3_N_3_ nanosheet to the protein adsorption event, we calculated the interaction energies, including vdW, Coulomb, and total energies, between the carbon/nitrogen of C_3_N_3_ and the protein as shown in Table S2. The results show that the carbon atoms on C_3_N_3_ present a larger contribution (i.e*.*, stronger vdW, Coulomb, and total energies) to the C_3_N_3_-protein adsorption than the nitrogen counterpart. In addition, we also calculated the binding free energy between the HP35 and the C_3_N_3_ surface via the MM/PBSA method^49^ as shown in Table S3. We noted that the vdW energy had a higher ratio in the binding free energy, which supports the previous result, as shown in Fig. 4b. In addition, the vdW energy bore the major contribution to the binding free energy between the HP35 and the C_3_N_3_ surface. The entire binding free energy showed a negative value, indicating that the binding of the HP35 protein onto the C_3_N_3_ nanosheet was energetically favorable.

**Figure 5:**
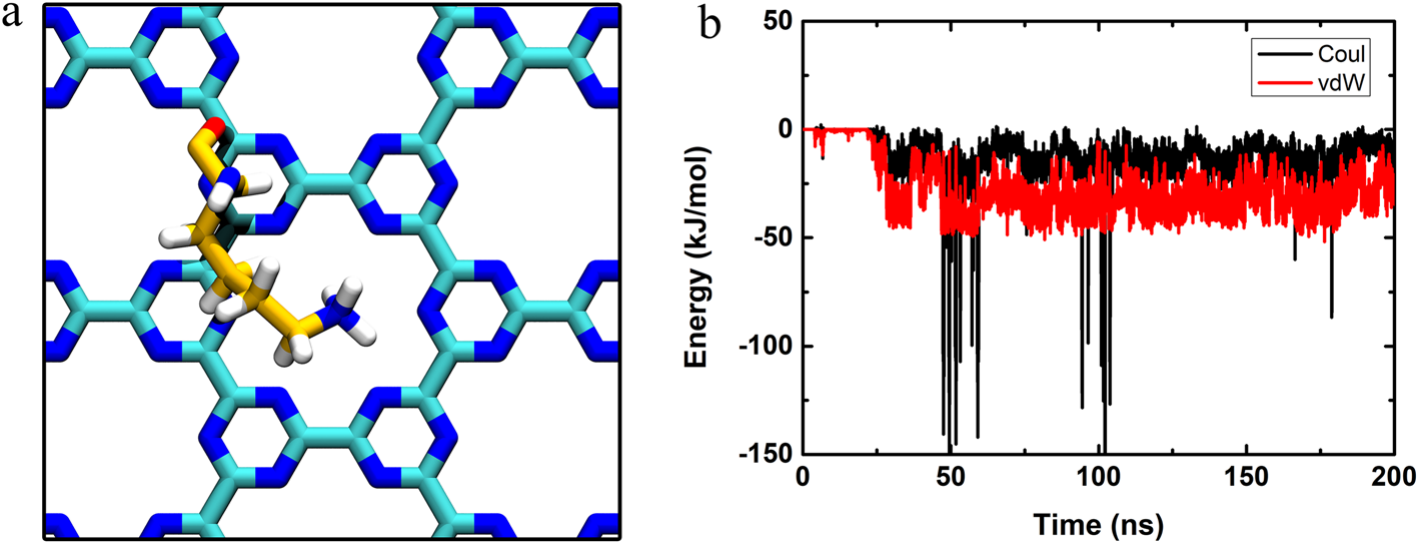
The specific interaction between a basic residue and the C_3_N_3_ hole. (**a**) Snapshot of the Lys71 residue binding to the C_3_N_3_ hole. The carbon, nitrogen, oxygen, and hydrogen atoms were depicted by yellow, blue, red, and white sticks, respectively. (**b**) Interaction energies, van der Waals (vdW) and Coulomb (Coul) energies, between Lys71 and C_3_N_3_, are shown.

We noticed that there were some sharp drops in the values of the Coulombic energy at some time points as illustrated in Fig. 4b. These changes in Coulombic energy were attributed to the specific interaction between the positively charged residues and the negatively charged nitrogen-rich hole on the C_3_N_3_ (Fig. 5), resembling the interaction profile between the C_2_N material and a protein^41^. Visual analysis indicate that indicates that Lys71 was stably attracted by the hole of the C_3_N_3_ stably, with its positively charged sidechain amino group (NH_3_^+^) pointing to the hole in the C_3_N_3_ structure. Later interaction energies between Lys71 and C_3_N_3_ exhibited similar profiles with sharp decreases in the Coulombic energy value reappeared around 50 ns and 100 ns, originating from the same interacting encounters as those in Fig. 4b. We also used MM/PBSA method to calculate the binding free energy of the Lys71 and C_3_N_3_. This binding free energy (Table S4) is a negative value, indicating that this adsorption of Lys71 over the pore is energetically favorable.

**Figure 6:**
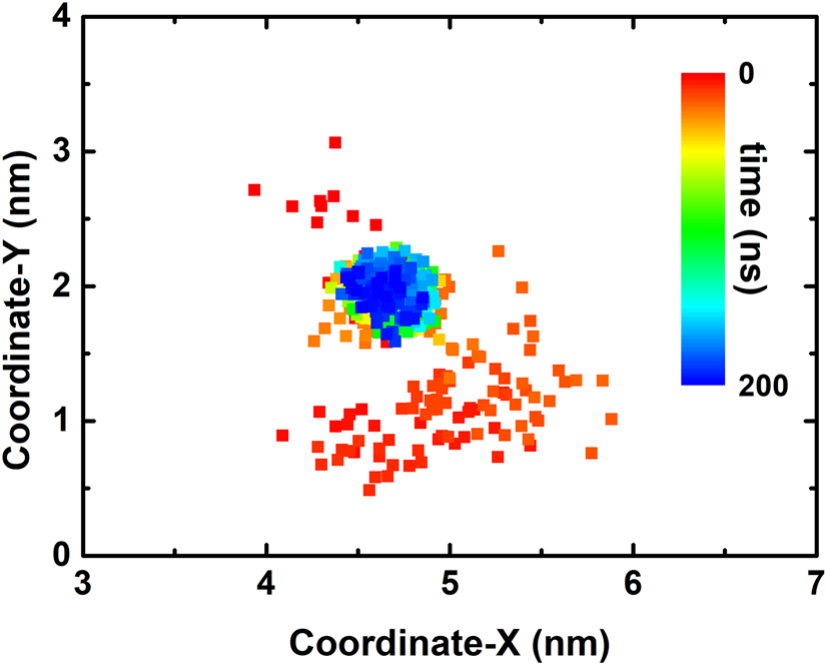
Tracking of the center of mass (CoM) of the HP35 projecting onto the X–Y plane (C_3_N_3_ surface) in the trajectory of sys1.

**Figure 7:**
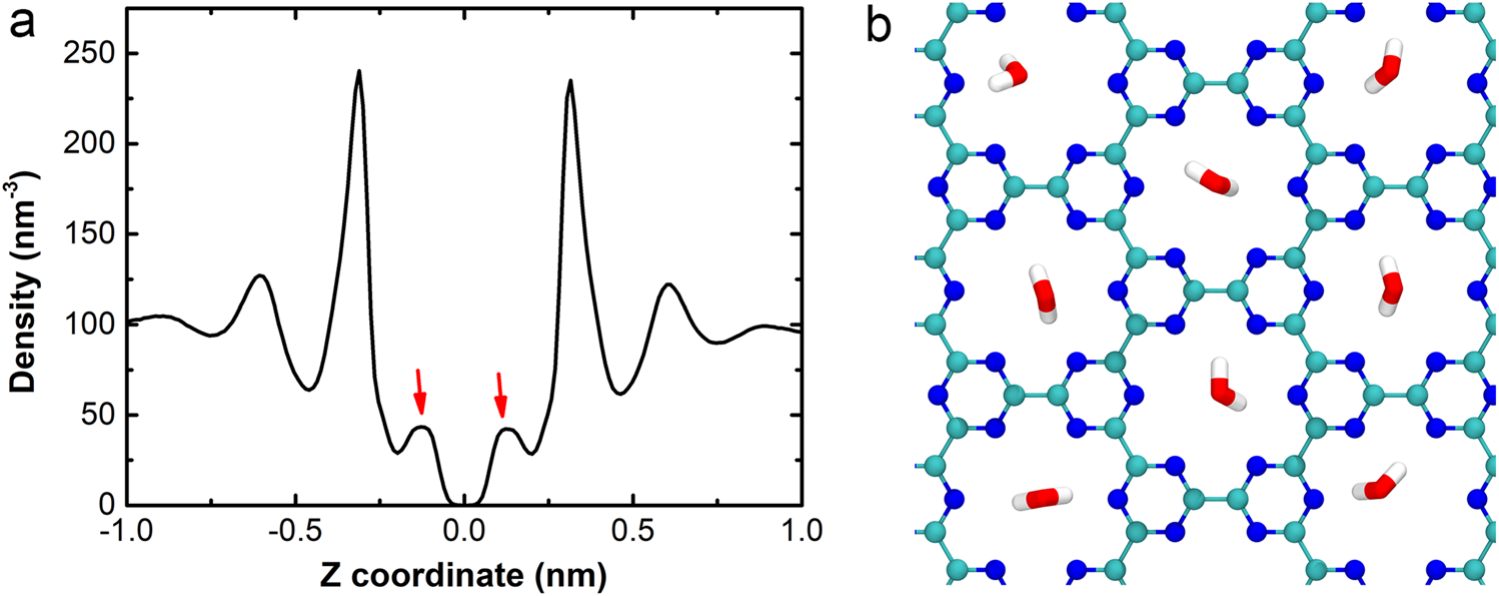
Water distribution on the C_3_N_3_ nanosheet. (**a**) Axial distribution of water density on C_3_N_3_ nanosheet. The unit of density is water number per nm^3^. The special water layer near the C_3_N_3_ was indicated by red arrows. (**b**) The conformation of the special water layer is denoted by (**a**). The water molecules are shown by red (oxygen) and white (hydrogen) sticks whereas the C_3_N_3_ nanosheet is displayed by ball-and-stick model.

Furthermore, we traced and project the center of mass (CoM) of the protein onto the X–Y plane (i.e., C_3_N_3_ surface) as shown in Fig. 6. It was clearly shown that the HP35 CoMs remain located at. one position, implying that was no lateral displacement of this on C_3_N_3_. This limited migration resulted in the protection of the HP35 structural integrity as described above, similar to a previous study on a different nanomaterial^41^. To further validate if the above specific binding profile between the basic residues of the protein and the C_3_N_3_ holes was responsible for the lack of lateral migration of the biomolecule, we performed an additional simulation where the atomic charges of the C_3_N_3_ structure were set to zero. Figure S6 illustrated the CoM track of the HP35 mapping onto the non-charged C_3_N_3_ surface. Interestingly, the same behavior of the protein, that is, the lack of lateral migration upon surface binding, was still observed. This result strongly suggests that the electrostatic interactions were not the main driven factor linked to the observed restrained binding pattern of the protein but that the vdW interactions alone were enough to induce the binding event. Therefore, the holes on the C_3_N_3_ structure played a dominant role to restrain the protein binding through the generation of high free energy barriers to hinder the free protein movement, as previously observed for the C_2_N nanosheet case^50^. Lastly, analysis of the surface water distribution on the C_3_N_3_ structure (Fig. 7) demonstrated that the surface holes tend to aggregate many water molecules (with one water molecule located nearby a hole), which generated a special water layer in the proximity of the C_3_N_3_ material. In addition, to further confirm the specific water layer formation on the C_3_N_3_ nanosheet, we conducted an additional simulation of the system comprising only a hydrated C_3_N_3_ nanosheet in the absence of protein and ions. Then, the water around the C_3_N_3_ nanosheet was analyzed as shown in Fig. S7. Clearly, in this system, the waters still form a specific water layer, which supports the finding when the protein is present and adsorbed onto the surface. This water layer is stabilized mainly by the electrostatic force and hydrogen bonds (Fig. S8). This water layer might also contribute to the restrained binding behavior of protein by forming a steric hindrance to prevent the lateral displacement of the protein, which resembled the results from our previous study regarding the dsDNA binding to the 2D C_2_N nanosheet^50^. In specific, the C_2_N nanomaterial also includes abundant holes on its surface structure, wherein each hole is comprised of six nitrogen atoms. Due to the inherent electron transfer from carbon to nitrogen, these holes displayed a negative charge center, which strongly attract water molecules. When the dsDNA was adsorbed on the C_2_N surface, these tightly adsorbed waters serve as obstacles that hinders the lateral movement of dsDNA on the C_2_N surface. Furthermore, by integrating the reports regarding the interaction between protein and other carbon nitrides (including C_3_N_4_^40^, C_2_N^41^, and C_3_N^42^), we have found that the porosity is a fundamental structural characteristic that delineates the interaction mechanism of biomolecules on these types of nanostructures. Specifically, the porous carbon nitrides (e.g., C_3_N_4_, C_2_N, and C_3_N_3_) comprise abundant ordered pores, which are uniformly encircled by some nitrogen atoms. These nitrogen atoms form negatively charged centers that attract both positively charged residues and water molecules; such interaction prevents the lateral movement of protein. In fact, the lateral movement of the protein on the 2D nanomaterials is critical to unfold the protein structure, for instance, the fast lateral shift of the HP35 on the non-porous C_3_N (like graphene) causes the severe structural denaturation of the HP35 protein^42^. To sum up, the restricted protein binding on the C_3_N_3_ nanosheet was mainly attributed to the inherent porous structure of the C_3_N_3_ nanosheet as well as the water aggregation in the proximity of the surface.

## Conclusion

In summary, we explored the adsorption of the HP35 prototypical protein onto the C_3_N_3_ nanosheet and the corresponding structural consequences of the biomolecule to evaluate the potential bio-effect of the recently synthesized C_3_N_3_ nanomaterial. Our results showed that HP35 could maintain native 3D-structural integrity upon adsorption onto the C_3_N_3_ nanosheet regardless of the protein interface involved in the binding event via the analysis of several structural parameters including the hydrophobic core native contact, α-helical structures, RMSD, hydrogen bond, and Q value. The vdW interactions mainly mediated the adsorption event while the kinetic process suggested that HP35 was able to pack on the C_3_N_3_ rapidly in a stepwise fashion. Meanwhile, a specific binding between a positively charged residue and a negatively charged C_3_N_3_ hole led to sudden declines in the Coulombic energy values during the entire simulation. Further simulations revealed that the non-disruptive binding of HP35 may be attributed to the restriction of the lateral migration of the protein on the C_3_N_3_ structure, which was derived from the surface porous topology and the steric hindrance of surface water. Our results provide insights into the effects of the novel C_3_N_3_ nanomaterial on protein structure, which will be central for the future biomedical applications of such nanomaterial.

## Methods

The C_3_N_3_ surface utilized in our simulations was a 7.4 × 6.4 nm^2^ nanosheet, comprising 648 carbon and 648 nitrogen atoms. The 35-residue chicken villin headpiece subdomain protein (HP35, PDB code: 1YRF)^51^ was selected because of its general properties associated with common globular proteins despite its small size^44–47^. Two initial simulation systems were built to probe the potential structural influence of the C_3_N_3_ surface to the HP35 as shown in Fig. 1b,c, of which HP35 was placed at an initial distance of ~ 1.5 nm from the C_3_N_3_ surface. The HP35 protein was rotated by 180° with its different sides facing the C_3_N_3_, yielding two systems as shown in Fig. 1b (defined as sys1) and Fig. 1c (defined as sys2). Each system was dissolved in a 0.15 M NaCl solution to simply mimic a physiological environment.

All simulations were carried out with the GROMACS software package^52^ using the CHARMM36 force field^53^. The parameters of C_3_N_3_ were obtained according to the protocol of previous studies^50,54^. We calculated the charges of a C_3_N_3_ model (Fig. S9) by quantum mechanics (QM) using Gaussian 09 at the HF/6-31G* level and parameterized using the RESP method, by which the atoms inside the black circle were chosen to extract the atomic charges of carbon (0.75 e) and nitrogen (− 0.75 e). The VMD software^55^ was used to analyze and visualize the simulation results. The force field details of C_3_N_3_ can be found in Table S1. The TIP3P water model^56^ was adopted to treat the water molecules since it is widely used to investigate the interaction between nanomaterials and biomolecules^57–61^. The temperature was maintained at 300 K using a v-rescale thermostat^62^ and pressure was kept at 1 atm using semiisotropic Berendsen barostat^63^ (only applied at the direction perpendicular to the C_3_N_3_ nanosheet). To avoid the “artificial collapsing” of nanosheets with their mirror images due to the limited size of the simulation box (which was due to the limited computational resources), the C_3_N_3_ nanosheet was fixed throughout the simulation process. The long-range electrostatic interactions were treated with the PME method^64^, and the van der Waals (vdW) interactions were calculated with a cutoff distance of 1.2 nm. All solute bonds associated with hydrogen atoms were maintained constant at their equilibrium values with the LINCS algorithm^65^, and water geometry was also constrained using the SETTLE algorithm^66^. During the production runs, a time step of 2.0 fs was used, and data were collected every 10 ps. Each system was investigated for three independent 200 ns trajectories.

### Supplementary Information

Supplementary Information.

## Data Availability

The datasets used and/or analyzed during the current study available from the corresponding author on reasonable request.
