# Supplementary material for: Moderate binding of villin headpiece protein to C3N3 nanosheet reveals the suitable biocompatibility of this nanomaterial
Source: Sci Rep. 2023 Aug 23;13:13783. doi: 10.1038/s41598-023-41125-1 (PMC10447452; doi:10.1038/s41598-023-41125-1)
Supplement: Supplementary file 1 — Supplementary Information. [file 41598_2023_41125_MOESM1_ESM.docx]

Table S1. Force fields for C_3_N_3_.

|  | ε (kJ/mol) | σ (nm) | Charge |
| --- | --- | --- | --- |
| N | 0.360 | 0.340 | -0.75 e |
| C | 0.711 | 0.325 | 0.75 e |

Table S2. Interaction energies (including vdW, Coulomb and total energies) between carbon/nitrogen of C_3_N_3_ and protein. The data were collected from the final 10 ns of the trajectory as shown in Figure 4.

|  | vdW energy (kJ/mol) | Coulomb energy (kJ/mol) | total energy (kJ/mol) |
| --- | --- | --- | --- |
| Interaction between carbon of C_3_N_3_ and protein | -61.76±3.60 | -48.92±35.12 | -110.69±34.29 |
| Interaction between nitrogen of C_3_N_3_ and protein | -77.53±4.91 | 21.98±35.12 | -55.55±35.46 |

Table S3. Binding free energy between HP35 and C_3_N_3_. The data are calculated from the last 5 ns.

|  | Energy (kJ/mol) |
| --- | --- |
| vdW energy | -154.87 |
| Electrostattic energy | -31.72 |
| Polar solvation energy | 12.12 |
| SASA energy | -13.53 |
| Binding energy | -188.00 |

Table S4. Binding free energy between Lys71 and C_3_N_3_. The data are calculated from the last 5 ns.

|  | Energy (kJ/mol) |
| --- | --- |
| Binding energy | -32.25 |


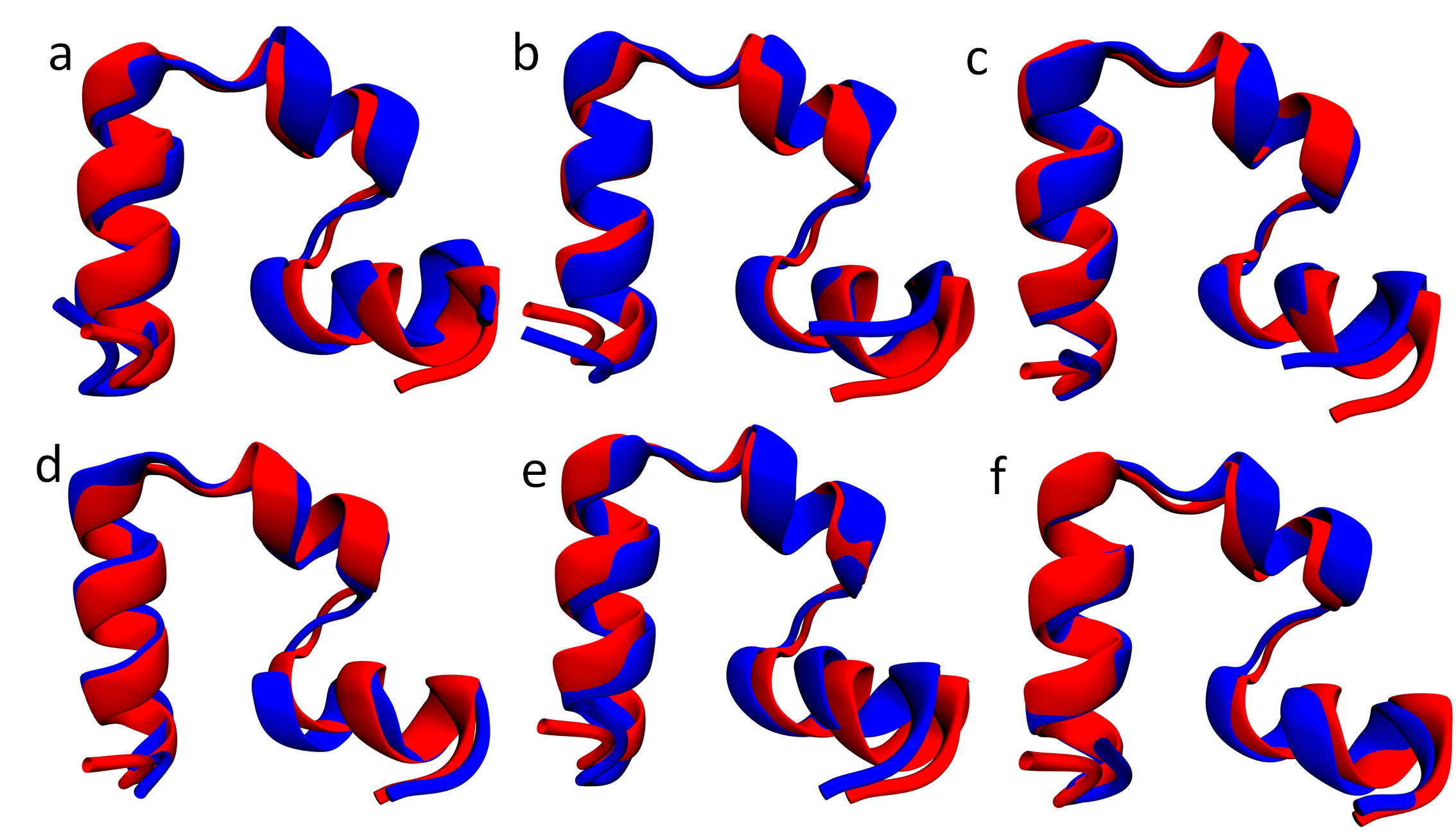


Figure S1. The structural alignment of HP35 at the original and final frames in the simulations of sys-1 (a-c) and sys-2 (d-f). Red: original HP35 structure. Blue: final HP35 structure.


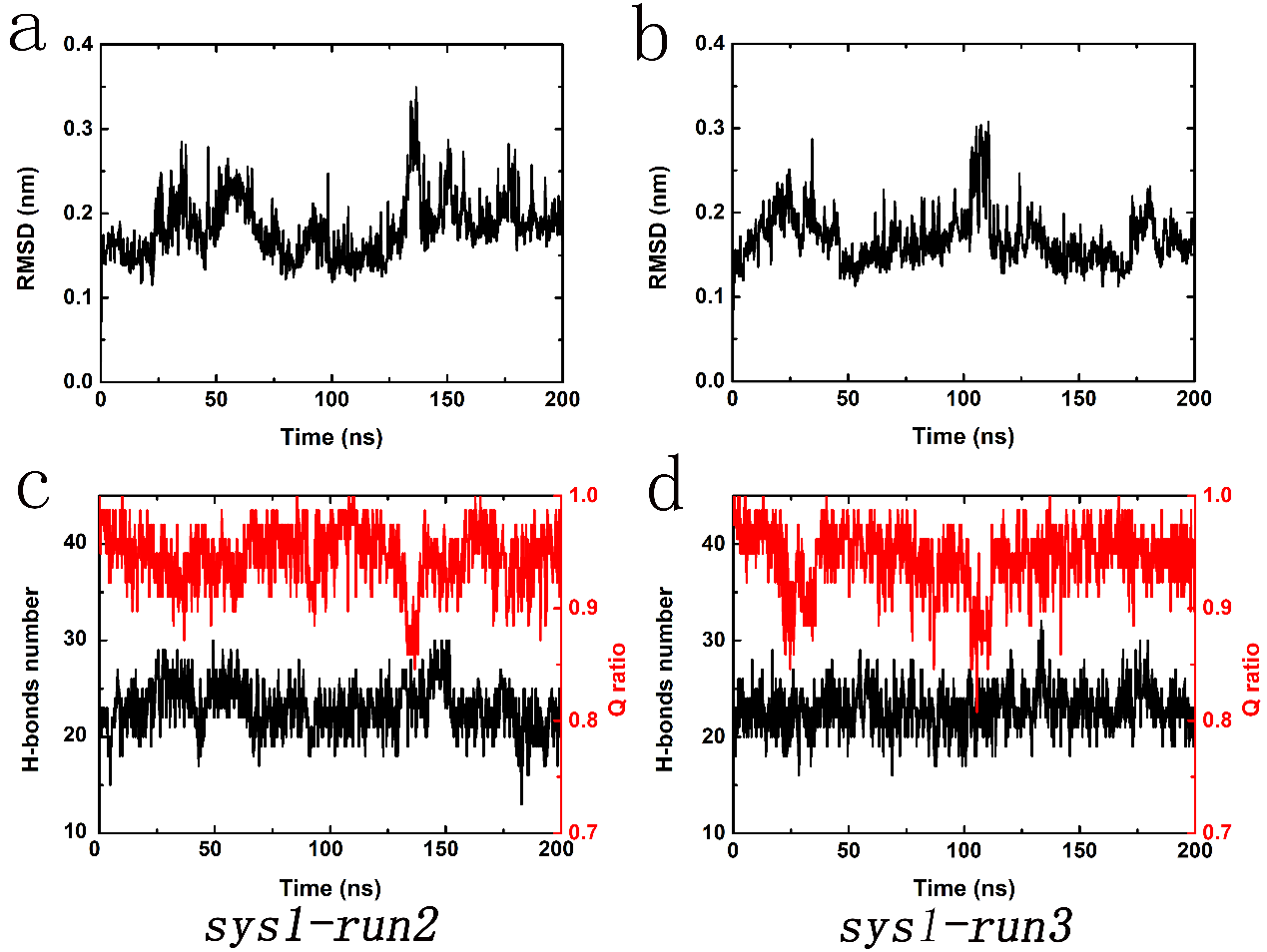


Figure S2. (a-b) The RMSD of heavy atoms of HP35 in other two trajectories of sys1. (c-d) The hydrogen bond (H-bond) and Q ratio evolutions of HP35 in other two trajectories of sys1.


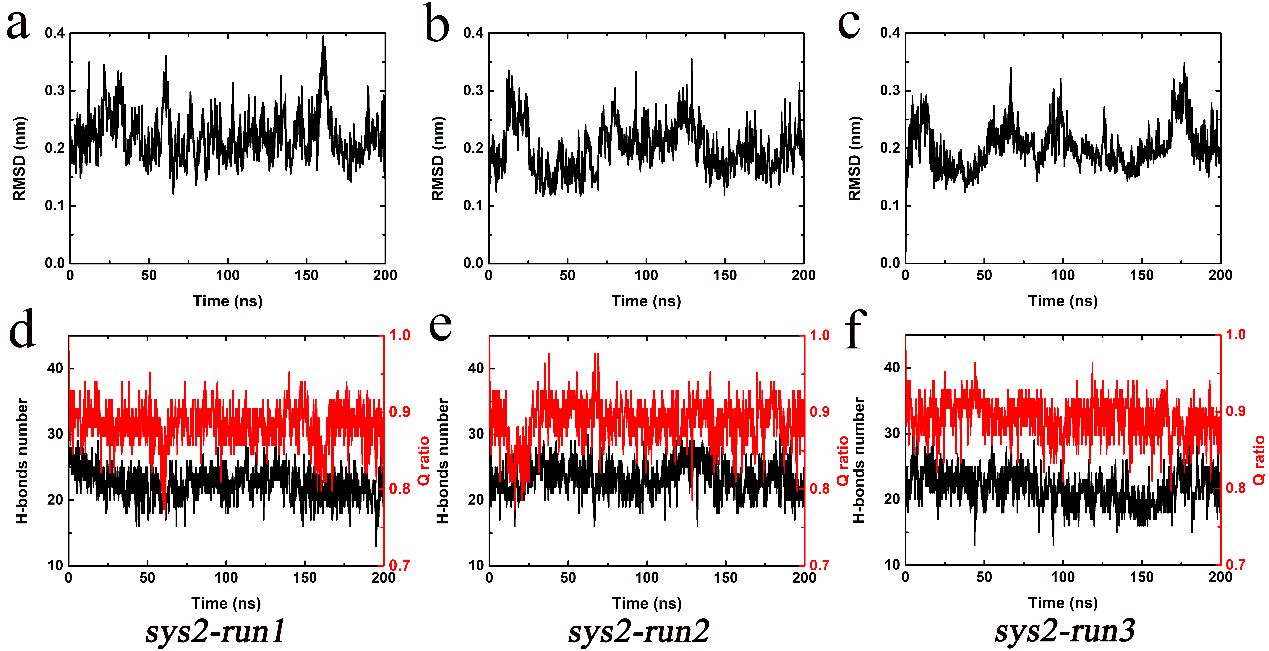


Figure S3. (a-c) The RMSD of heavy atoms of HP35 in three trajectories of sys2. (d-f) The hydrogen bond (H-bond) and Q ratio evolutions of HP35 in three trajectories of sys2.


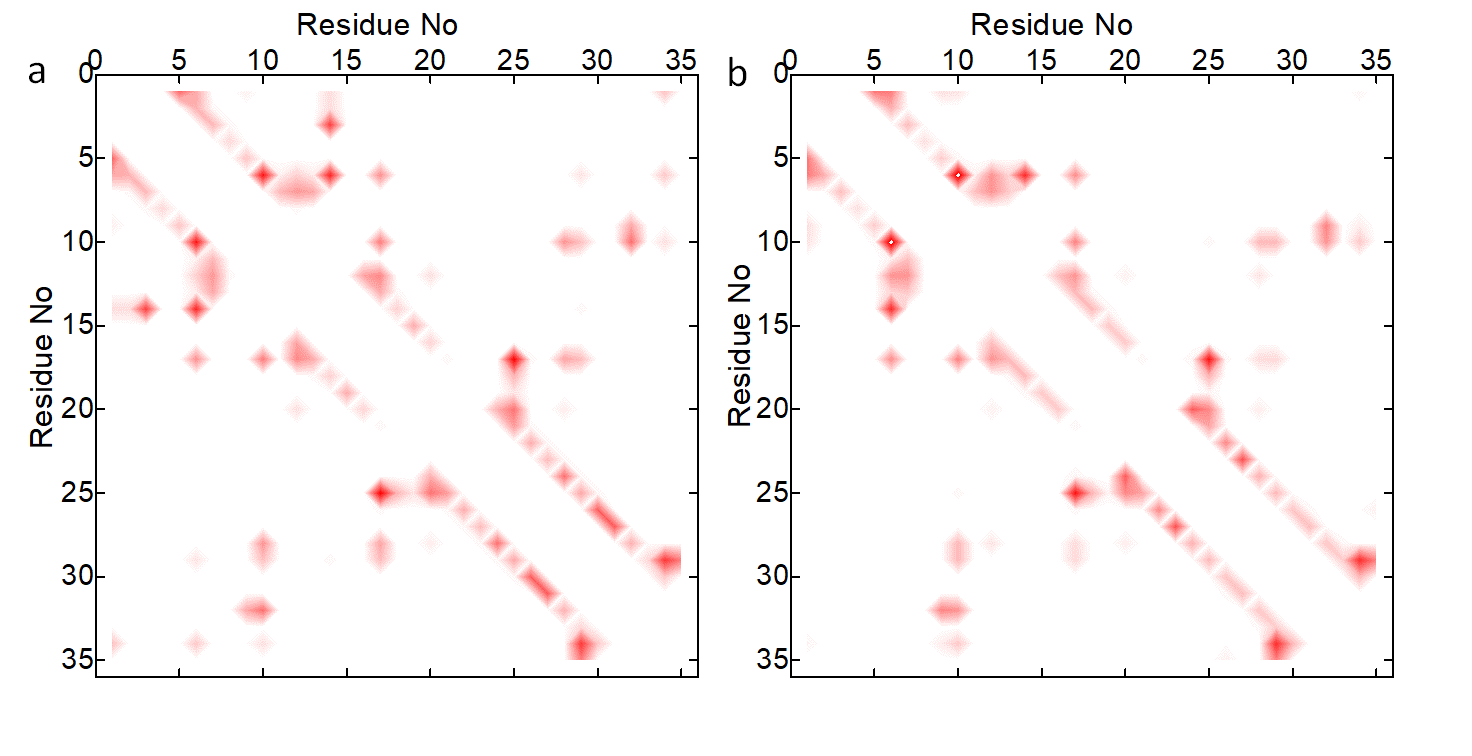


Figure S4. Two dimensional contact map within residues of HP35. (a) The contact map of HP35 at the first frame. (b) The contact map of HP35 at the final frame. The color ranging from white to red indicates the residue contact number increasing from zero to 64.


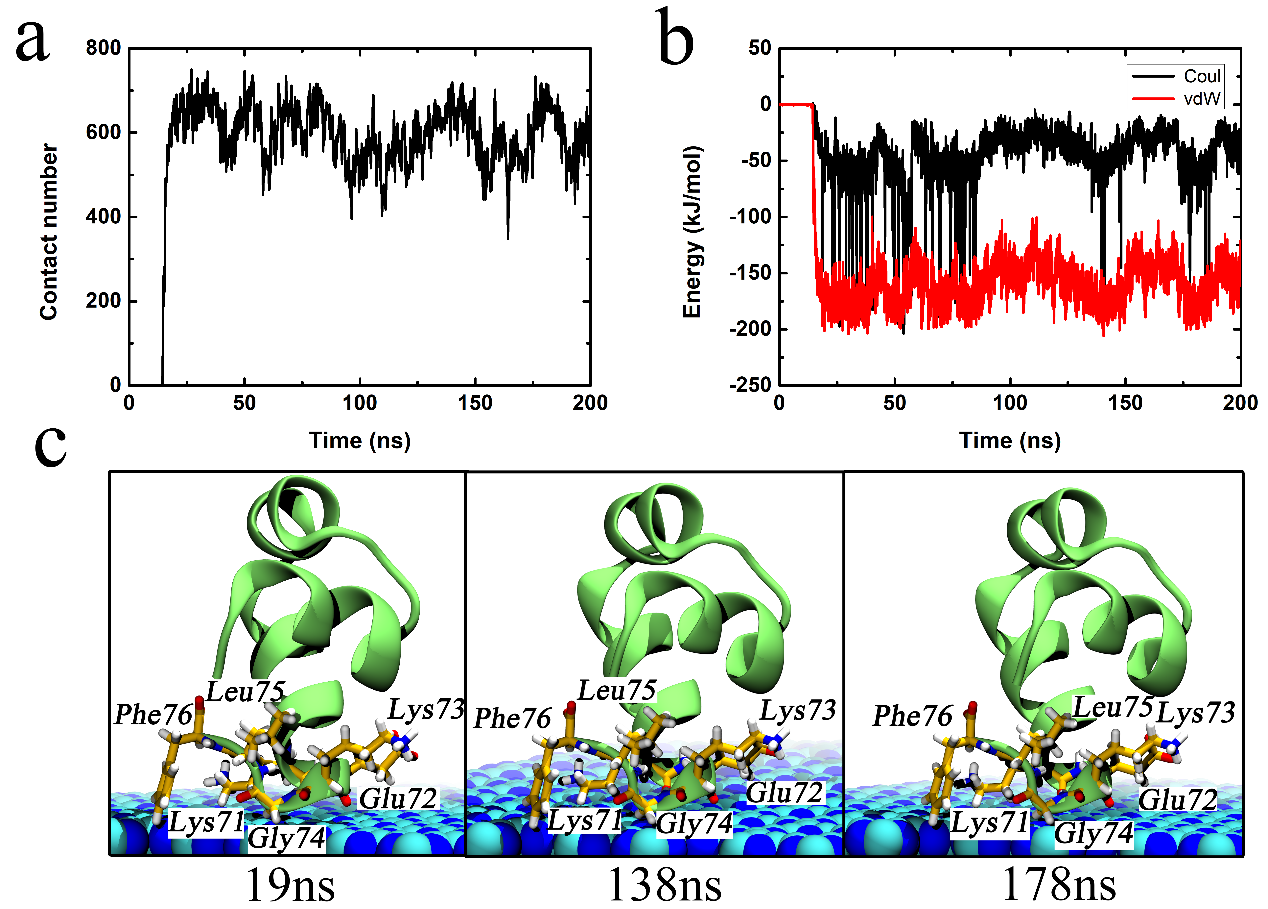


Figure S5. Interaction kinetics of HP35 binding to C_3_N_3_ nanosheet of sys2. (a) The contact number of HP35 binding to C_3_N_3_ nanosheet. (b) Interaction energies, including van der Waals (vdW) and Coulomb (Coul) energies, between C_3_N_3_ and HP35. (c) The binding conformations in some key time points. The key amino acids were displayed by sticks and labeled with their residue names.





Figure S6. The CoM track of HP35 binding to non-charged C_3_N_3_ nanosheet.


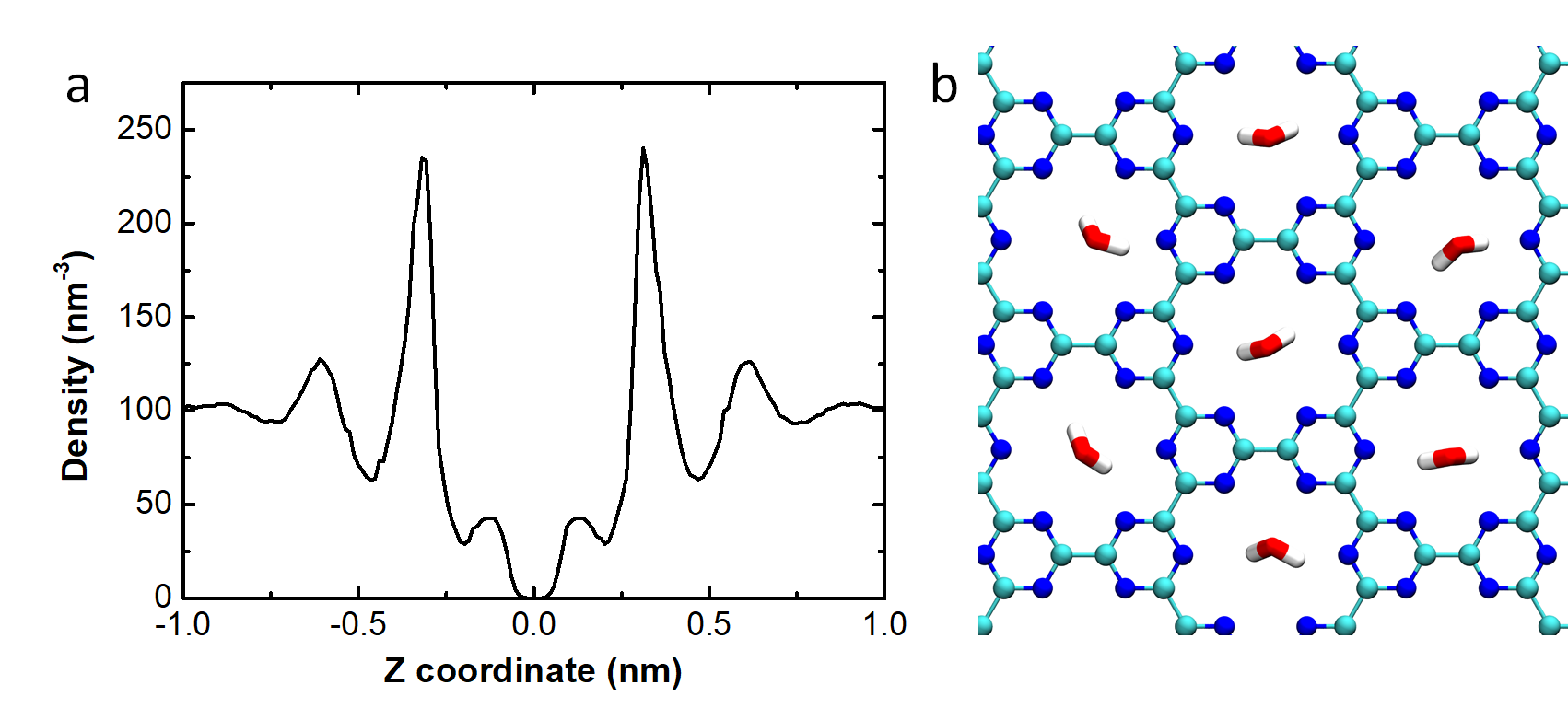


Figure S7. Water distribution on C_3_N_3_ nanosheet in the system comprising a C_3_N_3_ nanosheet and water. (a) Axial distribution of water density on C_3_N_3_ nanosheet. (b) The conformation of the special water layer. The water molecules were shown by red (oxygen) and white (hydrogen) sticks whereas the C_3_N_3_ nanosheet was displayed by ball-and-stick model.


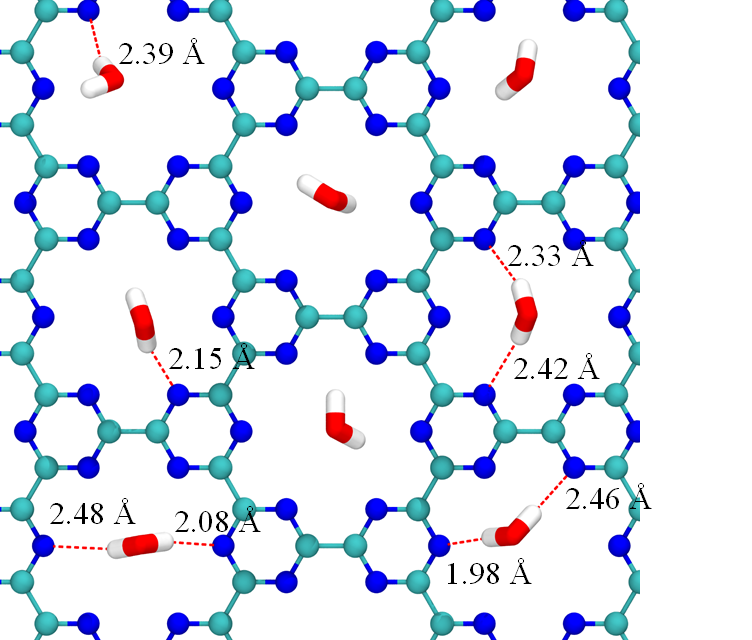


Figure S8. Hydrogen bonds formed between the interfacial waters and C_3_N_3_. The distances indicate the hydrogen bond lengths.


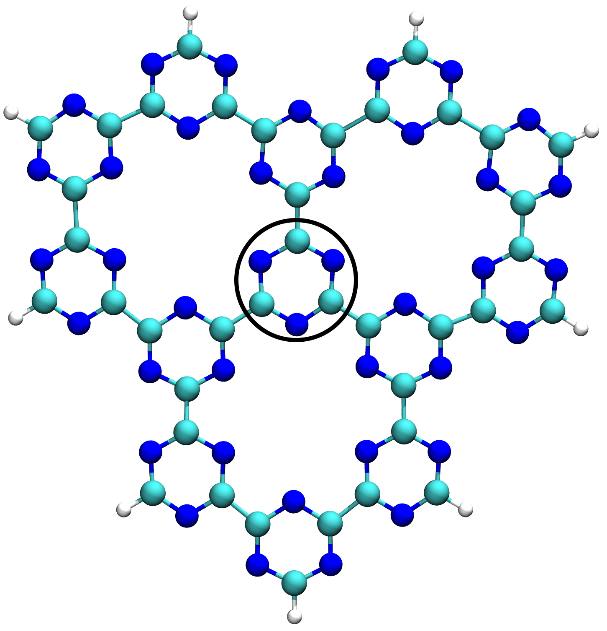


Figure S9. C_3_N_3_ flake in the QM calculations, the edge carbons were saturated with hydrogen atoms. The atoms inside the black circle were chosen to extract the charges of each atom.
